# Mapping and Functional Analysis of QTL for Kernel Number per Row in Tropical and Temperate–Tropical Introgression Lines of Maize (*Zea mays* L.)

**DOI:** 10.3390/cimb45050281

**Published:** 2023-05-18

**Authors:** Yuling Wang, Yaqi Bi, Fuyan Jiang, Ranjan Kumar Shaw, Jiachen Sun, Can Hu, Ruijia Guo, Xingming Fan

**Affiliations:** 1 Institute of Resource Plants, Yunnan University, Kunming 650504, China; 2 Institute of Food Crops, Yunnan Academy of Agricultural Sciences, Kunming 650205, China; 3 College of Agronomy and Biotechnology, Yunnan Agricultural University, Kunming 650500, China

**Keywords:** KNR, quantitative trait locus, genome-wide association study, candidate gene, heterotic pattern

## Abstract

Kernel number per row (KNR) is an essential component of maize (*Zea mays* L.) grain yield (GY), and understanding its genetic mechanism is crucial to improve GY. In this study, two F_7_ recombinant inbred line (RIL) populations were created using a temperate–tropical introgression line TML418 and a tropical inbred line CML312 as female parents and a backbone maize inbred line Ye107 as the common male parent. Bi-parental quantitative trait locus (QTL) mapping and genome-wide association analysis (GWAS) were then performed on 399 lines of the two maize RIL populations for KNR in two different environments using 4118 validated single nucleotide polymorphism (SNP) markers. This study aimed to: (1) detect molecular markers and/or the genomic regions associated with KNR; (2) identify the candidate genes controlling KNR; and (3) analyze whether the candidate genes are useful in improving GY. The authors reported a total of 7 QTLs tightly linked to KNR through bi-parental QTL mapping and identified 21 SNPs significantly associated with KNR through GWAS. Among these, a highly confident locus *qKNR7-1* was detected at two locations, Dehong and Baoshan, with both mapping approaches. At this locus, three novel candidate genes (*Zm00001d022202*, *Zm00001d022168*, *Zm00001d022169*) were identified to be associated with KNR. These candidate genes were primarily involved in the processes related to compound metabolism, biosynthesis, protein modification, degradation, and denaturation, all of which were related to the inflorescence development affecting KNR. These three candidate genes were not reported previously and are considered new candidate genes for KNR. The progeny of the hybrid Ye107 × TML418 exhibited strong heterosis for KNR, which the authors believe might be related to *qKNR7-1*. This study provides a theoretical foundation for future research on the genetic mechanism underlying KNR in maize and the use of heterotic patterns to develop high-yielding hybrids.

## 1. Introduction

Maize (*Zea mays* L.) is one of the world’s most important food, feed, and industrial crops and plays a major role in ensuring food security, socio-economic development, and alleviating the energy crisis. However, in recent years, corn varieties have experienced homogenization, leading to the erosion of diversity in the country’s corn gene pool. This has resulted in increased susceptibility to diseases and pests and reduced genetic resources for future crop breeding and improvement. To increase maize production, one viable approach is to conduct research on the temperate–tropical introgression germplasm and improve its grain yield (GY) per unit of land. Because kernel number per row (KNR) is a crucial factor affecting GY, increasing KNR in maize is a feasible way to enhance maize GY per unit of land.

As a critical yield-related trait, it is particularly important to decipher the genetic architecture and assess the performance of KNR. The formation of KNR is closely related to the differentiation and development of the female inflorescence in maize [1]. The number of kernels per ear of maize directly affects grain yield [2], which influences yield traits such as KNR, and by increasing KNR, the grain yield is effectively enhanced [3]. Several researchers have analyzed the yield traits from the perspective of their genetic effects and studied their inheritance patterns. Sabadin et al. indicated that KNR has a high heritability coefficient and correlation with total grain yield [4]. As quantitative traits are influenced and regulated by multiple factors, it is of great theoretical and practical importance to study their complex genetic mechanism to enhance the efficiency of genetic improvement, accelerate the breeding process, and develop the production potential of maize.

Quantitative trait mapping localizes the quantitative trait locus (QTL) of interest on the chromosome by analyzing the linkage between phenotypes and genotypes [5]. Genome-wide association study (GWAS) is a powerful tool [6] that investigates the causative loci using millions of single nucleotide polymorphisms (SNPs) [7]. SNPs mark the linkage disequilibrium (LD) between the trait and marker [8], allowing the researchers to further excavate the genes related to the targeted trait mutation. By combining GWAS analysis and QTL positioning, the KNR points were co-positioned, and the results could be complemented and verified [9].

Currently, with the development of molecular marker technologies, genetic localization using GWAS and QTL analysis has been widely used in molecular breeding [10,11,12,13,14]. This has helped reveal the genetic mechanisms of many quantitative traits, such as kernel row number, growth period, KNR, etc., and has facilitated the genetic improvement of these traits [15]. Genes such as inflorescence gene *EAD1* [16], *Thick tassel dwarf1* (*td1*) [17], *fasciated ear 2* (*fea2*) and *clavate 3/embryo surrounding region-related7* (*cle7*) [18], *compact plant 2* (*ct2*) [19], *fasciated ear3* (*fea3*) [20], *ramose locus2* (*rel2*) [21], and *fon2-like CLE protein 1* (*fcp1*) [22] can regulate the development of spikelet pair meristems (SPMs) and spike meristems (SMs). The restriction of inflorescence meristem (IM) proliferation affects panicle morphology [23,24], which further influences the formation of KNR. Numerous researchers have used different materials to locate different QTLs and genes associated with KNR in various environments [25,26,27]. For example, Liu et al. used introgression lines to fine-map the KNR main effect QTL *qKNR6* (within 198 kb) on chromosome 6, which codes for Ser/Thr protein kinase. Further studies determined that *Zm00001d036602* is a pathogenic gene of *qKNR6*, which controls the variation of KNR [28]. Zhang et al. used an F_2:3_ population to locate a main effect QTL *qKN* (within 480 kb) controlling KNR located between bnlg1360-umc1645 marker on chromosome 10 under different phosphorus concentrations [29]. Zhan et al. finely localized a kernel number per ear-related site *KNE4* (within 440 kb) and predicted that the candidate gene encodes a long-chain acyl Co-A synthetase that was involved in plant growth and development processes [30,31]. An et al. used two ILs to perform transcriptome and regional association analysis and found three SNPs located in three DEGs (*Zm00001d013277*, *Zm00001d015310,* and *Zm00001d015377*) that were closely related to KNR [32]. Zhang et al. detected multiple QTLs related to KNR using a diploid population of B73 × Mo17, among which *qKNR8* was the main effect locus [33]. Luo et al. used GWAS analysis to locate an ear length (EL) gene, *YIGE1*, which increases EL and kernel number per row (KNR) by regulating the size of the female IM [34]. Chen et al. identified a QTL *qKM4.08* encoding the retrotransposon subunit ZmVPS29 associated with variation in maize kernel morphology, and over-expression of this gene resulted in higher KNR and yield per plant [35]. Overall, these studies provide crucial insights into the genetic structure of KNR in maize.

Developing tropical and subtropical germplasm resources has been the key to creating core germplasm and solving problems in the seed industry. In this study, three parental inbred lines were used, which exhibited rich genetic variation and had definite yield advantages. One of the parental lines, the tropical germplasm CML312, developed by the International Maize and Wheat Improvement Center (CIMMYT), is a tester and is widely used for maize breeding due to its positive combining ability. Another parental line, the backbone line Ye107 derivative, is a selfed line bred in southwest China and has been used by breeders to develop a series of hybrids. Additionally, the temperate and tropical introgression maize variety TML418 also displays strong heterosis with other elite lines. In this study, TML418 and CML312 were used as female parents and crossed with a Ye107-derived line (common male parent) to produce two RIL populations of F_7_ generation with a total of 399 RILs, which showed wide genetic variation for KNR to conduct bi-parental QTL mapping and GWAS analysis. The objectives of this study were to: (1) identify QTLs and SNP loci significantly associated with KNR in different environments in both populations; (2) further identify new candidate genes associated with KNR; and (3) investigate whether using the new heterotic pattern improves breeding efficiency.

## 2. Materials and Methods

### 2.1. Test Materials and Field Design

**Figure 1:**
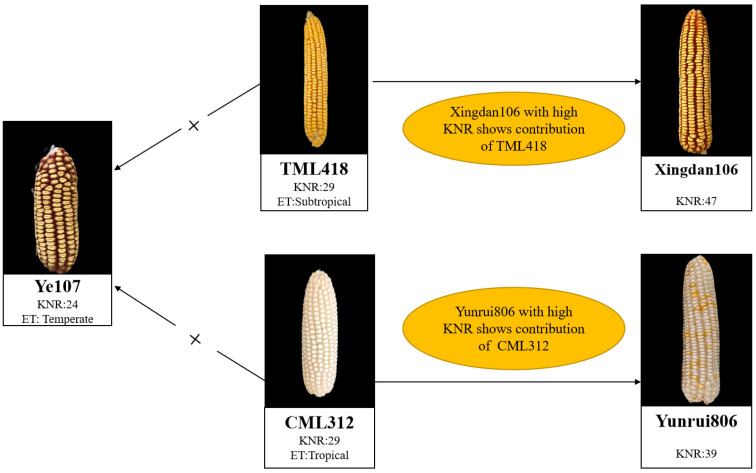
The breeding strategies using CML312, TML418, and Ye107. Each photo of the ear shows the line’s name, kernel number per row (KNR), and ecological type (ET). Lines with × represent a cross of parental inbreds; lines without × show the hybrid from their corresponding parental inbreds.

In the present study, a temperate–tropical introgression maize inbred line TML418 and a tropical maize inbred line CML312 (with abundant genetic variation for KNR) were used as female parents and crossed with the common male parent Ye107, which was a temperate inbred line with high seed yield (Table 1, Figure 1). The resulting F_1_s were self-crossed for seven consecutive generations by the single seed descend method to obtain two RIL subpopulations: pop1 (Ye107 × TML418) and pop2 (Ye107 × CML312), each consisting of 200 lines, for a total of 400 lines. Due to inbreeding depression and other environmental factors, only 399 RILs were obtained for genetic mapping studies. The RILs were planted in Dehong (DH) and Baoshan (BS) in Yunnan Province in 2018 and 2019, respectively. The experiments were set up in a randomized complete block design (RCBD) with two replications at each location. Each experimental plot consisted of 4 m long rows with 0.70 m inter-row spacing, 25 cm plant-to-plant spacing, and 14 plants per row. Standard agronomic practices were followed during the trials. After maturity and natural air drying, uniformly sized ears were selected from each family for the evaluation and recording of maize KNR data.

### 2.2. Phenotype Determination and Analysis

After preliminary processing of phenotypic data from two locations collected over two years with three replicates, normal distribution was estimated. Correlation analysis was performed using R software (V4.0.5) for KNR in different populations, years, and locations. Descriptive statistics were used to analyze the mean, standard deviation, variance, skewness, kurtosis, coefficient of variation, and range of variation of KNR. Broad sense heritability was calculated by referring to the method of Knapp et al. [36,37]:

(1) h2=σg2σg2+σge2e+σε2/re×100%,

where *σ*g^2^ refers to genetic variance, *σ*g*e*^2^ refers to variance due to environment x genotype interactions, *σε*^2^ refers to residuals, *e* refers to location, and *r* refers to the year [36]. As a quantitative trait, KNR is prone to environmental and other factors. *h*^2^ can help identify the phenotypic trait variation, and a larger *h*^2^ indicates that the trait is under stronger genetic control and less influenced by environmental factors.

### 2.3. DNA Extraction and Genotyping-by-Sequencing (GBS)

Genomic DNA was extracted from young maize leaves during the reproductive stage using the Hexadecyl trimethyl ammonium Bromide (CTAB) method [38,39]. After checking the quality and quantity, DNA was fragmented using ultrasound and end-repaired by adding adenosine (A) at the 3′ end. Sequencing adaptors were attached, and fragments of approximately 400 bp in size were enriched using magnetic beads. Subsequently, polymerase chain reaction (PCR) amplification was followed to build a sequencing library. After quality control, whole-genome resequencing was performed on the Illumina NovaSeq6000 platform in paired-end (PE) 150 mode. The sequencing reads were filtered, and BAM files were corrected using the GATK Best Practices process [40] to eliminate the duplexes introduced during sequencing. The error was resolved in the BAM comparison process through local re-comparisons of the indel regions. SNP and small InDel marker detection were performed after fixing the BAM files. The functional annotation of SNPs and InDels was carried out using ANNOVAR software based on the gene prediction information of maize reference genome B73 (RefGen_v4). Linkage analysis and genome-wide association analysis were conducted based on the identified SNP and InDel markers.

### 2.4. Construction of Genetic Linkage Map and QTL Mapping

The SNP data were filtered by setting the parameter MAF ≥ 0.05, as compared to the maize reference genome, B73 (RefGen_v4). Consecutive SNPs were examined in a sliding window of 15 SNPs in size. The ratio between the number of SNPs from the two parents was calculated in each window and used for genotype calling; as the window slides along the chromosome, recombination breakpoints were determined. The filtered high-quality SNPs were divided into ten linkage groups. Joinmap4.0 software was used to obtain markers within the linkage groups, estimate the genetic distance between adjacent markers, test the logarithm of odds (LOD) threshold by permutation tests 1000 times (LOD = 2.5), use the composite interval mapping (CIM) model to locate QTLs, and, finally, construct a linkage map of two RIL populations. The proportion of phenotypic variation explained (PVE) by each QTL was measured with the square of the correlation coefficient (R^2^).

### 2.5. Genome Wide Association Study

GWAS is an important tool for mining candidate genes for target traits by statistically examining the degree of association between phenotypes and high-density markers covering the entire genome. After Illumina NovaSeq6000 sequencing, the BAM files were processed, and GWAS analysis was performed using the GEMMA (https://www.xzlab.org/software.html, accessed on 15 November 2021) software [41]. The parameters were set to plink--indep-pairwise 50 5 0.2, −log10(P) > 5.0 and Mixed Linear Model (MLM) to adjust for population structure and individual kinship relationships [42]. The threshold was set at 0.05, and both Manhattan and QQ plots were generated.

### 2.6. Identification and Functional Annotation of Candidate Genes

Genes encoding proteins related to maize KNR were searched in the maize reference genome B73 (RefGen_v4) database and maize GDB (https://www.maizegdb.org/gbrowse, accessed on 15 November 2021). The screened genes were considered candidate genes related to maize KNR. The function of the candidate genes was annotated at NCBI (https://www.ncbi.nlm.nih.gov/, accessed on 15 November 2021) and in related websites and compared with the previous studies.

## 3. Results

### 3.1. Phenotype Analysis of KNR in Two RILs Populations

A phenotypic investigation of kernel number per row was conducted in two different locations, using two RIL populations, and data were collected. The coefficient of variation of the two RIL populations in the two environments ranged from 20.8% to 24.7% (Table 2), indicating significant differences between the samples, although little difference in the coefficient of variation was found in the same population [43,44]. The absolute values of KNR skewness and kurtosis in different environments were less than 1. The heritability (*h^2^*) in different environments ranged from 33.5% to 34.8%, which was low, indicating that KNR is easily affected by the environment [45]. Pearson correlation analysis [46] (*p* < 0.001) revealed a strong correlation between the two RILs populations in both DH and BS environments (r = 0.96/0.89). This indicates that the phenotype of KNR in the two populations was greatly affected by the environment, which was helpful in obtaining the localization of relevant molecular markers.

### 3.2. Linkage Analysis and QTL Mapping of KNR in Two RILs Populations

The genetic map of pop1 constructed in the present study comprises a total of 3287 SNP markers, with a length of 3003.474 centimorgan (cM). The average genetic distance between markers was 1.033 cM, with chromosome 2 having the highest number of SNPs (508) and chromosome 7 having the least number of SNPs (113). Chromosome 5 was the longest, with a genetic distance of 468.093 cM, and chromosome 3 was the shortest, with a distance of 144.936 cM (Figure S1, Table S1). The genetic map of the pop2 contained a total of 831 SNPs with a length of 3453.922 cM. The average genetic distance between markers was 1.1 cM, with chromosome 2 having the most SNPs (119) and chromosome 7 having the least (73). Chromosome 4 had the longest genetic distance of 806.611 cM, and chromosome 9 had the shortest length of 62.836 cM (Figure S2, Table S2 and Table 3).

**Figure 2:**
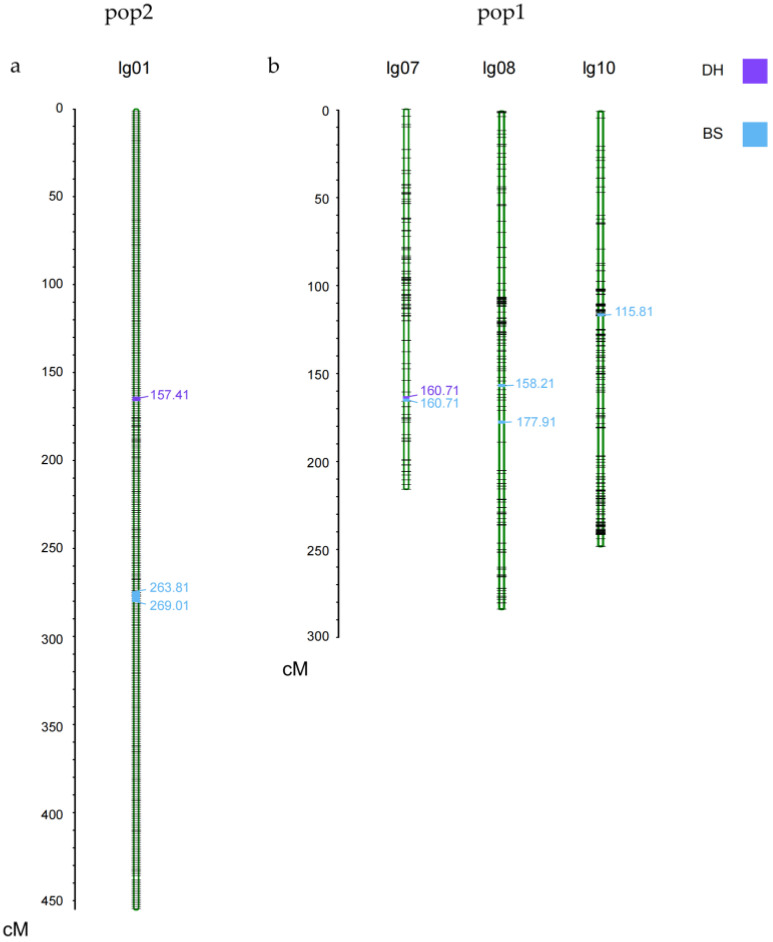
QTL localization distribution of pop2 (**a**) and pop1 (**b**).

QTL mapping and effect analysis were conducted for KNR in pop1 and pop2 in two different environments, filter SNP markers with deletion rates above 10% and loci with minimum allele frequencies less than 5%, with a LOD threshold set to ≥2.5. A total of four KNR QTLs were detected in pop1, including *qKNR7-1*, *qKNR8-1*, *qKNR8-2*, and *qKNR10-1* in two different environments, explaining 10.3%, 5.2%, 14.1%, and 5% of the phenotype variations, respectively (Figure 2, Table 4). Among them, the QTL *qKNR7-1*, identified on chromosome 7 in two different environments, had the highest LOD of 5.04 and the largest additive effect of 1.37. The remaining QTLs were detected only in the BS environment, and the additive effect values were negative. Three KNR QTLs, *qKNR1-1*, *qKNR1-2*, and *qKNR1-3*, were detected in pop2 in both environments (Figure 2, Table 4). All three QTLs were located on chromosome 1 and explained 9.5%, 6.6%, and 9.8% of the phenotypic variations, respectively. Among them, the QTL *qKNR1-3* had the largest LOD value of 3.21 and the largest additive effect of 1.54. The additive effect values of *qKNR1-1*, *qKNR1-2*, and *qKNR1-3* were all positive, indicating that these three QTLs had positive effects on KNR.

The Maize GDB website was used to predict candidate genes with QTL information, which were predicted to be possible candidates with respect to KNR. Since most of the QTL intervals were non-coding regions, only 2 genes were screened in the QTL intervals of the final genetic map of the two populations (Table 5).

### 3.3. Genome-Wide Association Analysis of KNR in Two RIL Populations

**Figure 3:**
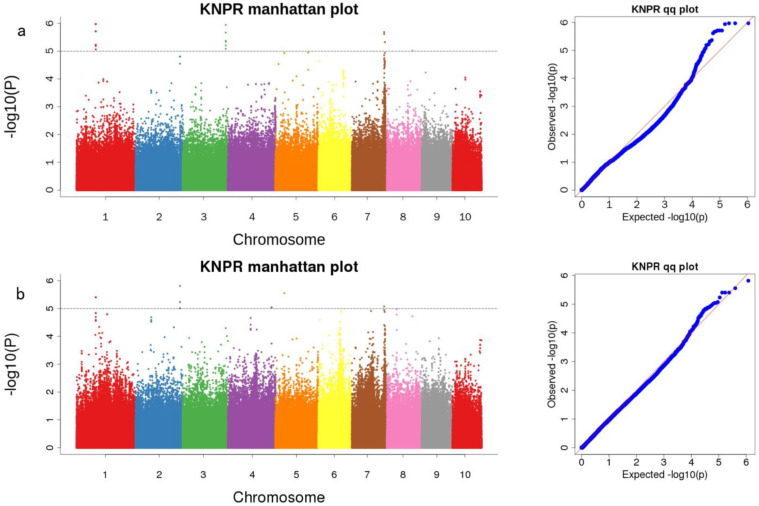
GWAS analysis for KNR in two RILs. (**a**) Manhattan plot and Q−Q plot of genome-wide association study for KNR in pop1 in two environments; (**b**) Manhattan plot and Q−Q plot of genome-wide association study for KNR in pop1 and pop2 in two environments.

538,697 valid markers (from the pop1) and 1,033,105 valid markers (both pop1 and pop2) were used to GWAS analysis (Figure 3) with MAF ≥ 5% and r^2^ < 0.2. A total of 5 SNPs significantly associated with KNR were identified in pop1, while 5 significant SNPs were identified in both populations. Additionally, eight and seven possible candidate genes were detected in the upstream and downstream regions spanning a 20 kb region of the above-mentioned loci in the two populations (Table 6). Notably, Snp-171585347, located on chromosome 7 in pop1, was repeatedly identified in two different environments.

### 3.4. Analysis of Consistent Sites Identified under Two Methods

Different authors have used various methods to define the overlapping regions between QTLs for the same trait, and the loci detected in common or those that overlapped under different environments were defined as consistent intervals [47]. In this study, we employed both bi-parental QTL mapping and GWAS analysis to compare the identified candidate QTLs, significant SNPs, and candidate genes from both approaches with the KNR QTLs available in public databases such as NCBI and Maize GDB. Our analysis revealed that QTL *qKNR7-1* identified through QTL linkage analysis and the marker interval of candidate SNP171585347 obtained through GWAS analysis are both located on chromosome 7 and have overlapping regions. Therefore, we considered these two intervals consistent intervals (Table 7). Based on the functional annotation of the candidate genes and previous studies, we identified three candidate genes (*Zm00001d022202*, *Zm00001d022168*, *Zm00001d022169*) associated with KNR formation within this consistent interval.

## 4. Discussion

### 4.1. The Phenotype of the KNR Is Strongly Influenced by the Environment

In this study, statistical analysis of KNR phenotypic data in two populations in different environments was conducted. The results revealed a high phenotypic diversity of KNR between the two populations. The heritability of KNR in both populations was found to be low, ranging from 33.5% to 34.8%. Furthermore, more than 89% r values between the environments indicated that the KNR phenotype was more influenced by the environment [48,49].

### 4.2. Three New Candidate Genes Associated with KNR Identified on Chromosome 7

The materials used in this study were tropical and subtropical, which are rich genetic resources compared to temperate germplasm. Additionally, different mapping methods and environments were employed in this study, resulting in the identification of novel QTLs different from the previous studies. QTL mapping and GWAS analysis identified a consistent locus, *qKNR7-1,* related to KNR, with a phenotypic variance of 10.3%. By sorting out the previously identified QTLs and SNPs associated with KNR on chromosome 7 and analyzing 400 yield-related QTLs, Wang et al. finally located the QTL *qKrow7-1* linked to KNR within the 150–200 cM interval on chromosome 7 [50]. Ma et al. used PHB47 and the American GEM germplasm YTL389ICA to construct the BC_3_F_4_ population and locate the KNR QTL *MQTLNP13* within the 162.81–165.51 cM of chromosome 7 [51]. Zhou et al. identified a QTL *qEL7.2* lined to KNR and EL within 271.30–273.80 cM on chromosome 7 [52]. Li et al. located the KNR QTL *qKr-7-1* at 35.58 cM on chromosome 7 [53]. Xiang et al. reported the QTL *Kn4* associated with KNR at 6.7 cM on chromosome 7 [26]. Liu et al. identified a QTL linked to KNR, *qKN7*, in the marker interval of bnlg1305-bnlg1808 at the N^+^ level [54]. An et al. identified a KNR QTL *q3xKR7* in the 94.91 cM interval between 146,007,035 and 147,108,062 on chromosome 7 [55].

In this study, the position of the KNR loci that were repeatedly located was compared with the previous studies mentioned above. No similar or overlapping intervals were found, indicating that *qKNR7-1* is a new candidate interval. Furthermore, *Zm00001d022202*, *Zm00001d022168*, and *Zm00001d022169* were identified as new candidate genes that could be associated with KNR. The reason for not locating the consistent loci in the pop2 population may be due to the short spike length of the germplasm used in the population development. As KNR is positively correlated with spike length, the presence of only a small number of effective markers in the genetic map of the pop2 could have resulted in the consistent QTL not being detected.

### 4.3. Functional Analysis of Three Candidate Genes Associated with KNR

In the present investigation, three candidate genes (*Zm00001d022202*, *Zm00001d022168*, *Zm00001d022169*) were screened and identified by searching NCBI, Maize GDB, and previous research reports. Among them, *Zm00001d02202* belongs to protein phosphatase homolog 2 of MYST family 1, which affects the transferase activity and participates in the metabolism of compounds containing nucleobases, biosynthesis, and protein modification processes. Furthermore, the expression level of this protein was higher in the early stages of the germ and in vegetative organs such as shoot apical meristems (SAM) and leaves. Significant differences in the expression of the spikelet-paired meristem (SPM) were found by changing the light conditions. The expression of this gene was related to the long photoperiod during the vegetative stages (VT) phase of the light-sensitive maize inbred line double M9, further associating it with the development of inflorescence in maize [56,57] and photosynthesis-related processes [58]. Additionally, the expression of this protein increased significantly when maize was subjected to biotic/abiotic stresses. This gene may also play an essential role in response to bacterial infection and participate in plant defense responses mediated by regulating histone acetylation levels [59].

*Zm00001d022168* gene encodes the AT hook-containing MAR binding 1-like protein, which is associated with protein degradation and degeneration [60]. This gene is highly expressed in nutritive and reproductive organs, such as shoot apical meristem, stems, and seeds. Furthermore, the expression of *Zm00001d022168* varies with changes in light duration, with the highest expression level found in the SPM tissues after 12 h of light exposure [61]. In maize, the function of this gene was annotated by a gene homology study against Arabidopsis, and this gene was associated with maize root morphology and growth rate [62]. In Arabidopsis, the AT-hook motif protein AHL22 regulates flowering initiation by modifying T chromatin at the flowering site and interacts with histone methyltransferase [63]. The AT hook-containing MAR binding 1-like protein (AHM1) is a component of the nuclear matrix of wheat, connecting the nuclear framework and MARs. Therefore, it was speculated that AHM1 may be involved in the damage repair process caused by high temperatures [64].

*Zm00001d022169*, also known as GRMZM2G381395 (*rpot1*), belongs to the DNA/RNA polymerase superfamily protein and is associated with reproduction, nucleobase-containing compound metabolism, biosynthesis process, and cellular and mitochondrial composition [65]. Its expression in the SAM tissue, stem, endosperm, seed, and other vegetative organs and reproductive organs was found to differ with changes in illumination time. In addition, gene expression studies in maize filaments revealed that *Zm00001d022169* was closely related to the pollen–pistil interaction [66]. This gene, known as male gametophyte defect 3 (*MGP3*) in maize, is homologous to an Arabidopsis gene. Homozygous mutation of this gene significantly delayed the growth of pollen tubes, affecting the growth of the ovule and endosperm. Although the mutation did not affect the formation of pollen grains, structural defects of mitochondria in pollen grains were related to the mutation of this gene [67]. By studying the differential expression of genes in early inflorescences of maize, it was discovered that genes such as *Zm00001d022169* contributed to maize heterosis and were related to the genetic control of the maize lateral branch [68].

Overall, this study screened and compared the functional genes located in the significantly associated SNP loci region and identified three important candidate genes that were expressed in SAMs and SPMs. The formation of KNR relies on the continuous differentiation of the female inflorescence meristem (IM) into SPM [69], SPM into spikelet meristem (SM), and SM into floral meristem (FM) [70,71]. This study identified three novel genes associated with KNR formation in maize for the first time and suggested that they could be new candidates for KNR-related genes. These findings provide a theoretical basis for further investigation into the genetic mechanisms underlying meristem organization, advancing the understanding of KNR development in maize, and breeding high-yielding auto-synthetic maize lines and hybrids.

### 4.4. Combining Molecular Breeding with Conventional Breeding Methods to Improve Breeding Efficiency

In maize breeding, the improvement of yield, quality, and disease resistance depends on the continuous enhancement, innovation, and utilization of germplasm resources used as the starting material. Heterosis, where a hybrid population shows superior performance to the parents, is attributed to the emergence of new beneficial alleles or variants in gene expression resulting from gene expression modification [72].

The EL is positively correlated with the KNR. The SNP in the putative *YIGE1* region acts on the promoter, thereby increasing gene expression and resulting in an increase in the hybrid’s EL and KNR [28]. In addition, W138 and Mo17 germplasms carrying the ideal allelic gene *qEL1.10* can significantly improve the EL, KNR, and ear weight (EW) of the hybrid population [73]. The breeding efficiency using the heterozygous dominance model is 21% higher than the efficiency of the traditional breeding model [74].

The three parental lines used in this study have already been utilized to produce many excellent maize varieties. CML312, a derivative of CIMMYT lines, is one of the best test varieties. Many varieties with disease resistance and stable yield were developed using CML312, such as “Xikang18” and “Longbai1”. The Ye107-derived line is the backbone of southwest China’s inbred lines. This line has produced several maize varieties with excellent quality, disease resistance, and stress resistance, such as “Ludan8”, “Dedan5”, “Baoyu8”, and “Yunrui8”. Yunrui8 is a leading variety of the Ministry of Agriculture of PRC bred by the present author’s team. With TML418 as the parental line, we developed high-yielding corn varieties such as “Xingdan106”, “Yunrui668”, and “Yunrui16”. In a previous study, we showed that the heterosis effect of plant height (PH) and ear height (EH) plays an important role in reducing the PH and EH of offspring hybrids and achieving high yields [7].

The variety “Xingdan106”, developed from the parental lines TML 418 and Ye107 (pop1), has a higher KNR value compared to traditional breeding varieties, with a density ranging from 3500 to 4200 plants per acre. Its seed output rate reached 81.05%. In 2022, its dry grain yield per acre in the maize demonstration area reached up to 1078.1 Kg, achieving a good yield per acre. The success of Xingdan106 has also promoted corn production in surrounding towns and villages. This success confirms that by using parents with high EL and KNR, hybridization can significantly improve the KNR value of the population, which is probably related to the *qKNR7-1* locus. Further validation is required to confirm the presence of this locus in the future. To advance systematically and shorten the breeding cycle, it is essential to combine molecular breeding with conventional breeding, thus, improving the efficiency of innovatively using maize germplasm resources.

## 5. Conclusions

In this study, two F_7_ RIL populations were constructed by crossing the co-parental Ye107 derivative with the inbred lines TML418 and CML312, respectively. A total of 399 RILs were subjected to KNR QTL localization and GWAS analysis using 4118 validated SNP markers. QTL mapping detected a total of seven QTLs related to KNR in both populations, and GWAS analysis identified twenty-one SNPs significantly associated with KNR. Among them, one high-confidence significant locus, *qKNR7-1,* was repeatedly detected, and three candidate genes (*Zm00001d022202*, *Zm00001d022168*, *Zm00001d022169*) related to KNR were screened within the 20 kb range upstream and downstream of the locus. All three candidate genes were associated with maize inflorescence development, and the authors further speculated that they were related to the development of maize KNR. The three candidate genes identified in this study were the first report showing possible relevance to KNR. This may provide a reference basis for future research on the genetic mechanism of KNR and cloning of KNR-related regulatory genes in maize. It also provides a theoretical basis for breeding high-yielding hybrids.

## Figures and Tables

**Table 1:** Information on the parental lines.

Parents	Pedigree	Heterotic Group	Ecological Type
Ye107	Derived from US hybrid DeKalb XL80	Reid	Temperate
CML312	S89500-F2-2-2-1-1-B*5-2-1-6-1	Non-Reid	Tropical
TML418	Derived from Monsanto hybrid in Thailand	Non-Reid	Subtropical

**Table 2:** Statistical analysis of KNR phenotype of two RILs populations.

Populations	Environment	Mean	Standard Deviation	Skewness	Kurtosis	Coefficient of Variation (%)	Rang of Variations	*h^2^* (%)	Population Heritability (%)	r
pop1	18DH	17.461	3.631	0.224	−0.415	20.8	10–27	33.6	33.6	0.96
	18BS	17.608	3.854	0.328	−0.217	21.9	9.6–28	33.5		
pop2	19DH	18.215	4.496	0.491	0.395	24.7	8–32.6	34.7	32.7	0.89
	19BS	17.564	4.321	0.261	0.175	24.6	7.5–31	34.8		

18DH represents the trials conducted in Dehong in 2018, and 18BS represents the trial conducted in Baoshan in 2018. 19DH represents the trials conducted in Dehong in 2019, and 19BS represents the trials conducted in Baoshan in 2019.

**Table 3:** Distribution of SNPs and genetic distance estimation in pop1 and pop2.

Mapping Population	Chromosome	Number of SNP Markers	Length of Chromosome (cM)	Average Distance (cM)
pop1	1	305	208.792	0.685
	2	508	324.439	0.639
	3	336	144.936	0.431
	4	413	315.664	0.764
	5	442	468.093	1.059
	6	421	384.379	0.913
	7	113	216.03	1.912
	8	197	282.949	1.436
	9	375	410.947	1.096
	10	177	247.245	1.397
	Total	3287	3003.474	-
	Mean	328.7	300.347	1.033
pop2	1	79	437.997	5.544
	2	119	606.246	5.095
	3	100	342.968	3.430
	4	142	806.611	5.680
	5	62	295.757	4.770
	6	81	309.359	3.819
	7	42	148.887	3.545
	8	79	213.109	2.698
	9	73	62.836	0.861
	10	54	230.152	4.262
	Total	831	3453.922	-
	Mean	83.1	345.392	1.100

**Table 4:** Positions and effects of KNR QTLs detected in two RILs populations.

Mapping Population	QTL	Chromosome	Position (cM)	Mapping Interval	LOD	Additive Effect	R^2^
pop1	*qKNR7-1*	7	160.71	170,843,056–171,585,347	5.04	1.37	0.103
	*qKNR8-1*	8	158.21	101,154,574–107,979,743	2.75	−0.91	0.052
	*qKNR8-2*	8	177.91	110,450,438–136,744,298	4.59	−1.51	0.141
	*qKNR10-1*	10	115.81	46,406,004–46,600,980	2.81	−0.89	0.050
pop2	*qKNR1-1*	1	157.41	122,555,185–174,447,829	2.57	1.48	0.095
	*qKNR1-2*	1	263.81	87,932,948–87,933,008	2.53	1.27	0.066
	*qKNR1-3*	1	269.01	82,649,009–87,932,982	3.21	1.54	0.098

**Table 5:** Candidate gene prediction of the QTLs.

Population	Chromosome	Position (cM)	Mapping Interval	Candidate Gene	Gene Annotation
pop1	7	165.94	172,759,694	Zm00001d022202	protein phosphatase homolog2
pop2	1	266.45	2,237,453	Zm00001d027300	protein PAIR1

**Table 6:** Distribution of significant SNP loci and candidate genes in the two RIL populations for KNR identified through GWAS.

Mapping Population	Marker	Chromosome	Mapping Interval	*p*-Value	Candidate Gene	Gene Annotation
pop1	Snp-99488079	1	99,467,951–99,508,106	5.97	Zm00001d030014 (dist = 63,542)	tetraspanin family protein
					Zm00001d030015 (dist = 45,616)	-
	Snp-224368029	3	224,347,842–224,388,029	5.94	Zm00001d044290	Beta-galactosidase 1
	Snp-167546055	7	167,526,037–167,566,055	5.68	Zm00001d021985	formin-like protein 13
	Snp-171585347	7	171,565,347–171,605,347	5.32	Zm00001d022168 (dist = 26,086)	AT hook-containing MAR binding 1-like protein [*Zea mays*]
					Zm00001d022169 (dist = 111,235)	RNA polymerase T phage-like 1
	Snp-132258909	8	132,238,909–132,278,909	5.01	Zm00001d010888 (dist = 11,763)	-
					Zm00001d010889 (dist = 54,452)	myb-like protein J
both	Snp-99488079	1	99,467,951–99,508,106	5.4	Zm00001d030014 (dist = 63,542)	tetraspanin family protein
					Zm00001d030015 (dist = 45,616)	-
	Snp-230569504	2	230,549,473–230,589,504	5.82	Zm00001d007391	-
	Snp-226862468	4	226,842,468–226,882,476	5.04	Zm00001d053342	-
					Zm00001d053345	-
	Snp-45947193	5	45,927,193–45,967,193	5.55	Zm00001d014421	Growth-regulating factor 6
	Snp-167546055	7	167,526,055–167,566,055	5.07	Zm00001d021985	formin-like protein 13

**Table 7:** Consistent sites detected in two different mapping approaches.

Marker	Chromosome	Position	Mapping Interval	Candidate Gene	Gene Annotation
*qKNR7-1*	7	160.71 cM	170,843,056–171,585,347	Zm00001d022202	protein phosphatase homolog2
Snp-171585347	7	171,585,347 bp	171,565,347–171,605,347	Zm00001d022168	AT hook-containing MAR binding 1-like protein
				Zm00001d022169	RNA polymerase T phage-like 1

## Data Availability

The data presented in this study are available on request from the corresponding author.

## Supplementary Materials

The following supporting information can be downloaded at: https://www.mdpi.com/article/10.3390/cimb45050281/s1, Figure S1: Genetic map of pop1, Figure S2: Genetic map of pop2, Table S1: Pop1 genetic map data, Table S2: Pop2 genetic map data.
