# Supplementary figures and images for: Mapping and Functional Analysis of QTL for Kernel Number per Row in Tropical and Temperate–Tropical Introgression Lines of Maize (Zea mays L.)
Source: Curr Issues Mol Biol. 2023 May 18;45(5):4416–30. doi: 10.3390/cimb45050281 (PMC10217162; doi:10.3390/cimb45050281)

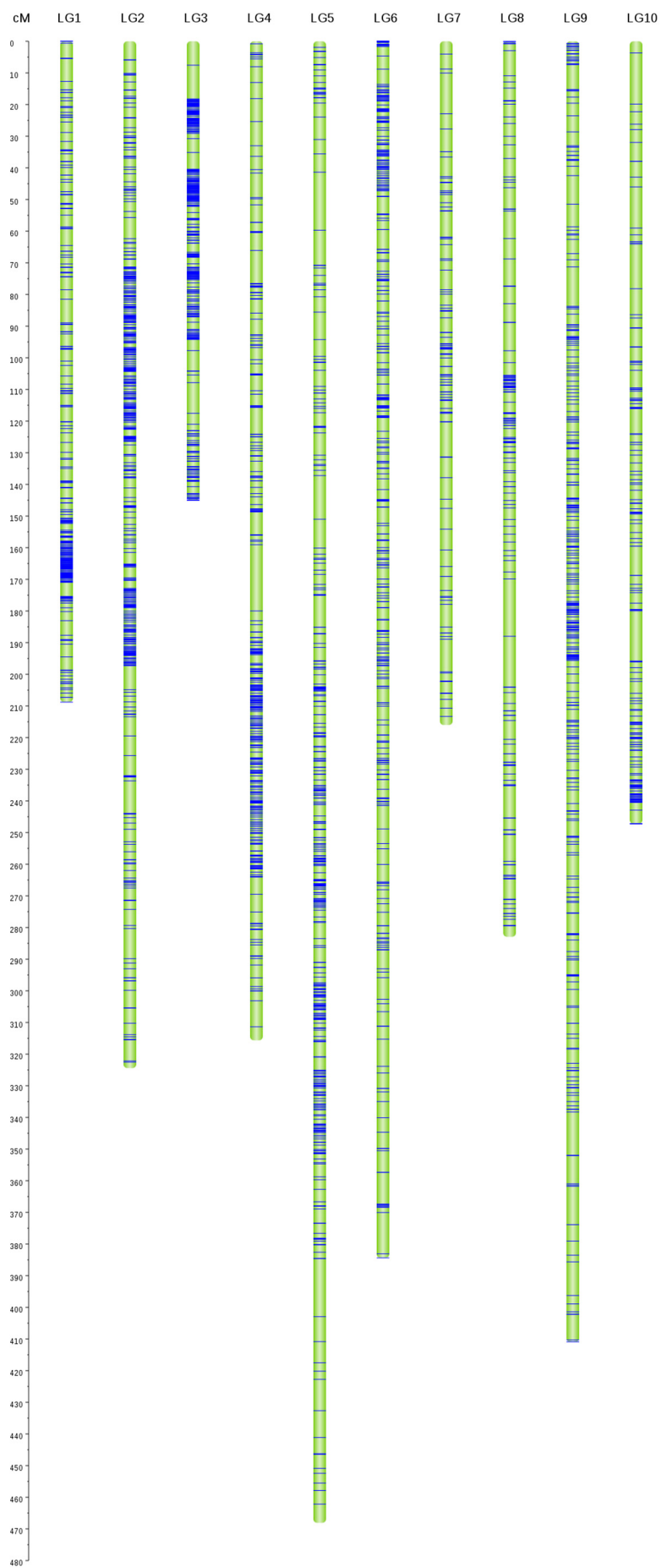

**Figure S1.** Genetic map of pop1.

Supplement: Supplementary file 1 [file cimb-45-00281-s001.zip › Figure S1.pdf]

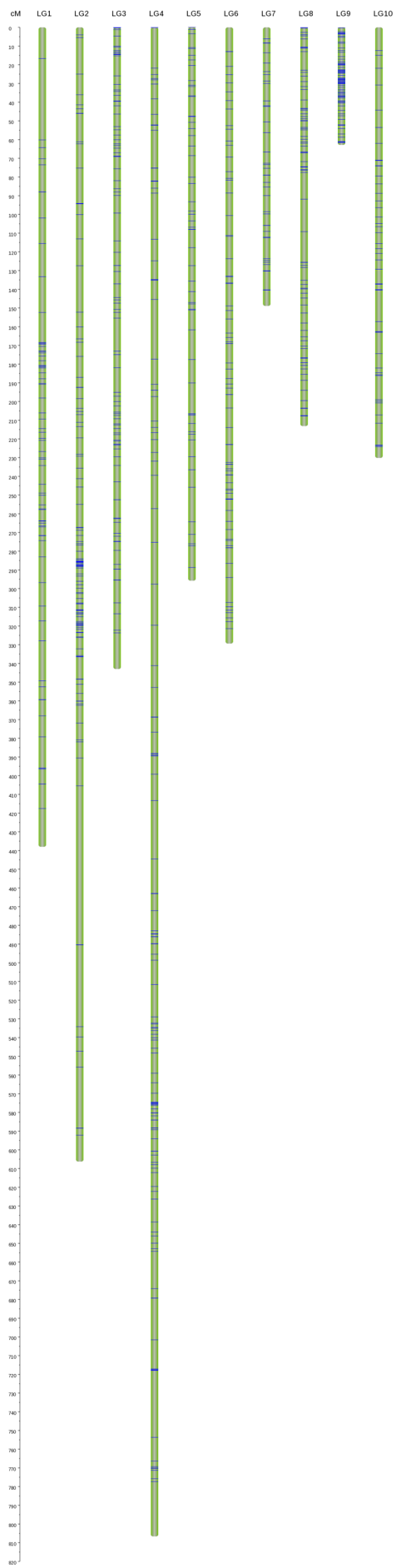

**Figure S2.** Genetic map of pop2.

Supplement: Supplementary file 1 [file cimb-45-00281-s001.zip › Figure S2.pdf]
